# Bioinspired Electronic White Cane Implementation Based on a LIDAR, a Tri-Axial Accelerometer and a Tactile Belt

**DOI:** 10.3390/s101211322

**Published:** 2010-12-10

**Authors:** Tomàs Pallejà, Marcel Tresanchez, Mercè Teixidó, Jordi Palacin

**Affiliations:** Department of Computer Science and Industrial Engineering, University of Lleida, Jaume II, 69, 25001 Lleida, Spain; E-Mails: tpalleja@diei.udl.cat (T.P.); mtresanchez@diei.udl.cat (M.T.); mteixido@diei.udl.cat (M.T.)

**Keywords:** blind, white cane, white stick, LIDAR, accelerometer, tactile

## Abstract

This work proposes the creation of a bioinspired electronic white cane for blind people using the whiskers principle for short-range navigation and exploration. Whiskers are coarse hairs of an animal's face that tells the animal that it has touched something using the nerves of the skin. In this work the raw data acquired from a low-size terrestrial LIDAR and a tri-axial accelerometer is converted into tactile information using several electromagnetic devices configured as a tactile belt. The LIDAR and the accelerometer are attached to the user’s forearm and connected with a wire to the control unit placed on the belt. Early validation experiments carried out in the laboratory are promising in terms of usability and description of the environment.

## 1. Introduction

Blindness is frequently used to describe severe visual impairments with or without residual vision. The extent of vision loss is described using an international scale [1]. According to the World Health Organization (WHO) in 2006 there were approximately 314 million people around the world whose vision is impaired [2]. Of this number, there are 45 million blind people and the most frequent causes are cataracts (47%), glaucoma (12%), age-related macular degeneration (9%), corneal opacity (5%), diabetic retinopathy (5%), childhood blindness (4%), trachoma (4%), onchocerciasis (1%) and others (13%).

The traditional methods to help blind and partially sighted people to move around on foot are the white cane (or white stick) and the guide dog [3] that is very expensive due the training process and maintenance. The white cane is a long stick that operates as a contact tool. It is useful to detect obstacles, stairs and curbs in the walking path while the eco of the typical sound originated when contacting with the floor gives average volumetric information to the user and also alerts other people in their walking path. Currently, there are many centers around the world researching and developing devices known as Electronic Travelling Aids (ETA) in order to improve the everyday life of blind people.

### 1.1. Related work

ETA devices are based on the use of some sensors to obtain data around the user and extract relevant positioning information. This information can be obtained by attaching ultrasonic [5,6] or infrared sensors [7] to the user’s body, using an external mobile robot (such as an artificial guide dog replacement) [8,9], replacing the white cane with a single laser rangefinder [10], and using vision based systems to convert visual data into an alternate rendering modality that will be appropriate for a blind user [4].

Alternatively, the environment can be modified to facilitate blind displacement. For example, large flat areas can include cheap magnetic parts in the ground to generate different sidewalks that can be detected using magnetic sensors [12]. The image acquired by a single camera can be processed to detect standard landmarks as pedestrian crossings [11]. The new Radio Frequency IDentification (RFID) targets can be used to identify different elements of the environment, such as the door of a bus [13], products in a supermarket [14], or also sidewalk marks [15]. The frequency and power of the signal emitted by several antennas distributed around a building [16] can be used to guide a blind through the rooms.

Specifically, the whisker effect has been largely used for short-range navigation and exploration in robotics [17]. Currently, mobile robot navigation is mainly based on the whisker effect using non-contact laser based LIght Detection And Ranging (LIDAR) sensors to find long-range paths and detect obstacles [18]. For example, in [19], a LIDAR was used as a whisker to guide an autonomous humanoid robot in urban areas and, in [20], several LIDARs and cameras were used to develop a semi-autonomous vehicle to be operated by the visually impaired.

### 1.2. Contribution of This Work

This work proposes the creation of an electronic white cane inspired in the effect of the whiskers, coarse hairs of an animal's face that tells the animal that it has touched something using the nerves of the skin. In this proposal the physical hairs are replaced by some laser beams generated by a general purpose terrestrial LIDAR combined with a tri-axial accelerometer. The LIDAR and the accelerometer are mounted over the user’s wrist to obtain relevant information about the environment and generate tactile stimuli on the skin of the waist using several electromechanical devices attached to a special belt. This proposal specifically aims to avoid the use of sound information or the use of tactile devices in the hands to increase the degree of freedom of the user while performing such everyday activities as walking. The promising early validation results obtained combined with the continuous reduction in the price and size of current terrestrial LIDARs enables future commercial electronic white cane developments.

## 2. Materials and Methods

This section describes the volunteers involved in this work and the necessary instrumentation to implement and validate the proposed electronic white cane device.

### 2.1. Test Subjects

The validation experiments developed in this work were carried out by volunteer Ph.D. students and laboratory assistants with no visual impairment but blindfolded while using the device. Future improved implementations of the proposed electronic white cane will be tested by actual blind users.

### 2.2. Instrumentation

The bioinspired electronic white cane is based on the combined use of two main sensors: A LIDAR and a tri-axial accelerometer and some electromechanical actuators to generate tactile information in the skin of the user.

#### 2.2.1. LIDAR

**Figure 1.**
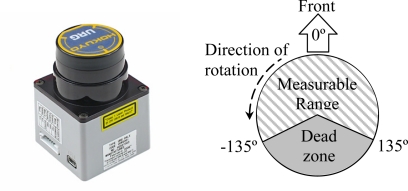
Laser URG-04LX (left) and measurable range (right).

The laser used in this work is the URG-04LX (Figure 1, left) manufactured by HOKUYO AUTOMATIC CO. [21]. The main physical characteristics of this laser are its small size (50 × 50 × 70 mm), low weight (142 g), and small power consumption (5V, 0.9 A at start up and 0.5 A in normal operation) enabling the development of portable and battery operated devices such as the proposed electronic white cane. The angular working range of this device is from −135° to 135° (Figure 1, right) providing a sequence of 769 distances with an angular resolution of 0.35° at 10 scans per second. According to the manufacturer, the range of measurable distances are from 60 to 4,095 mm with an accuracy of ±10 mm in the range from 60 mm to 1,000 mm and ±1% of the measurement in the remaining range. However, the real range rises to 6,000 mm with such big, highly reflective targets as walls and doors. The main objective of this sensor in the electronic white cane is to scan and get information of the environment around the user.

#### 2.2.2. Accelerometer

The accelerometer used in this work is the LIS3L02AL, a general-purpose accelerometer developed by STMicroelectronics [22]. This is a small (5 × 5 × 1.6 mm), low-power, 3-axis linear capacitive accelerometer that measures acceleration on three axes, x, y and z. Acceleration is measured in the range of ±2 g with scaling of approximately 200 counts per g and refreshed approximately 100 times per second. The main objective of this sensor in the electronic white cane is to measure the tilt of the LIDAR attached to the forearm on the 3 axes.

#### 2.2.3. Electromechanical device

**Figure 2.**
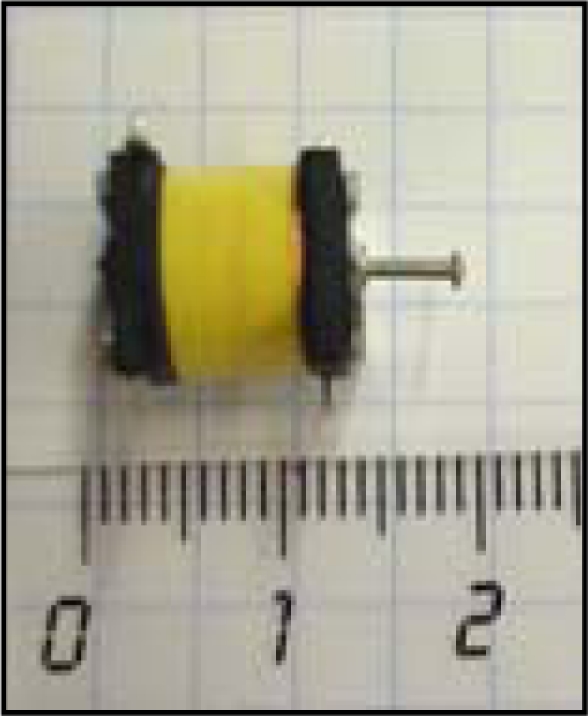
Miniature push solenoid with the stroke released (scale in cm).

The electromechanical device used in this work is a low cost miniature (10 × 12 mm) push solenoid developed by BLP Components Ltd. [23] with reference 45-320-620-623 (Figure 2). The solenoid weighs 4.6 grams and is operated at 5V/32mA with a maximum stroke of 4 mm when active. The main objective of this miniature solenoid is to generate pressure information on the skin of the user’s waist. The number of solenoid used defines the effective number of hairs of the equivalent whisker.

## 3. Electronic White Cane Proposal

**Figure 3.**
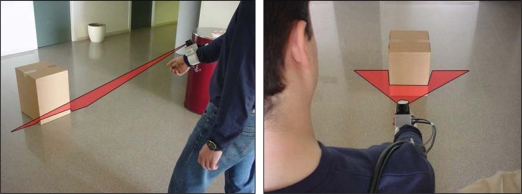
Image of the element that carries the LIDAR and the accelerometer of the electronic white cane and a representation of the scan of an obstacle in the walking path of the user.

The electronic white cane is made up of two portable elements. The first element is placed between the wrist and the forearm and carries the LIDAR and the accelerometer (see Figure 3). The second element is the tactile belt worn on the user’s waist, also carrying the batteries and the system control unit. In this prototype, the batteries and the control unit were replaced by a standard laptop PC with a wire connection to the sensors and actuators in the electronic white cane.

**Figure 4.**
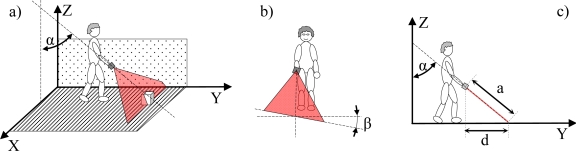
Representation of the electronic white cane operating in floor mode: **(a)** vertical inclination angle α, **(b)** horizontal orientation angle β, and **(c)** distance, d, to the line defined by the intersection of the scan plane and the floor.

**Figure 5.**
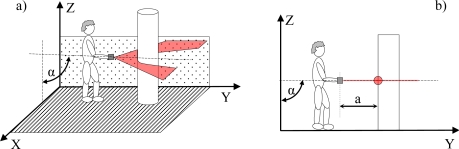
Representation of the electronic white cane operating in frontal mode: (a) general representation and (b) distance to the main obstacle.

It is proposed to operate the electronic white cane in two different modes that depend on the vertical inclination of the forearm measured by the accelerometer: the floor (Figure 4) and frontal mode (Figure 5). The electronic white cane operates in floor mode (LIDAR oriented to the floor) (Figure 4) if the inclination of the forearm of the user [α angle in Figure 4(a)] is close to 45° and its horizontal orientation [β angle in Figure 4(b)] close to 0°. In these conditions the LIDAR covers a range from 1 to 1.5 m in front of the user [Figure 4(c)], an improvement over the 75 cm covered by the conventional white stick. The electronic white cane operates in frontal mode (Figure 5) if the inclination of the forearm [α angle in Figure 5(a)] is close to 90° (parallel to the floor) and then no objects are expected in the scans acquired (any object detected generates a tactile description). The floor mode is expected to be used while walking. The frontal mode is expected while the user is stopped in a fixed position exploring the surrounding environment with the movement of the forearm.

### 3.1. Estimating Forearm Orientation

The estimation of the forearm orientation is used to establish the operating mode of the electronic white cane. The information acquired by the accelerometer are the relative acceleration levels in the *x*, *y* and *z* axis. The vertical inclination α [Figure 4(a)] and the horizontal orientation β [Figure 4(b)] of the forearm can be estimated from the raw data of the accelerometer using equations (1):

(1) $$\begin{array}{l} \alpha =-{\text{tan}}^{-1}(x/z)\\ \beta ={\text{tan}}^{-1}(y/z) \end{array}$$

**Figure 6.**
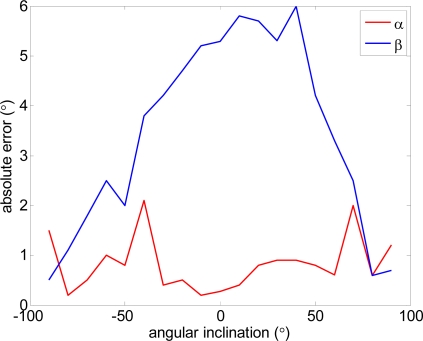
Absolute error in the estimation of the vertical and horizontal inclination.

Figure 6 compares real and estimated vertical and horizontal angles computed from the raw data of the accelerometer without any previous calibration. The measurement experiments were carried out on a stable platform. In a first experiment the vertical inclination α was changed from −90° to 90° while the horizontal inclination was 0°. In a second experiment the horizontal inclination β was changed from −90° to 90° while the vertical inclination was 0°. The results of Figure 6 show that the absolute error in the estimate of α was lower than 2° and in the estimate of β lower than 6°. These errors levels were considered acceptable for the application proposed; any further reduction would require specific calibration procedures applied to the accelerometer of each electronic white cane.

**Figure 7.**
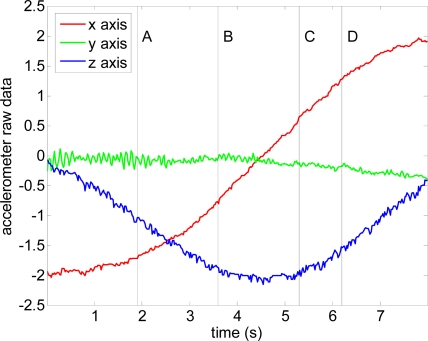
Acceleration data obtained when raising the forearm. Table 1 show positions A, B, C and D.

Figure 7 shows a case example with the complete evolution of the relative accelerations measured by the accelerometer when a static user raises his forearm from vertical inclination 0° to +160° (raw value 0 corresponds to no acceleration).

Table 1 shows real and estimated vertical inclination α corresponding to the four intermediate forearm positions defined in Figure 7. Table 1 also shows the image of the position of the forearm and a graphical representation of the three relative accelerations measured by the accelerometer. Despite the natural oscillations induced in the forearm during this movement, the angular error obtained in several similar experiments was always lower than 4°.

**Figure 8.**
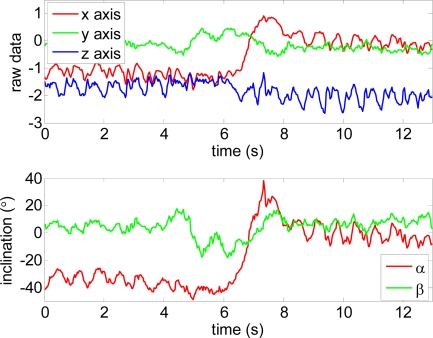
Raw sensor data for each axis and sensor inclination estimate during operation with the electronic white cane.

Finally, Figure 8 shows raw sensor data for each axis and the estimate of the vertical and horizontal inclination angles α and β. The data of the figure corresponds to three different user actions and gestures: (i) time from 0 to 5 s, normal walk with the forearm at approximately 40°, (ii) time from 5 to 8 s, the user stops and turns 180°, (iii) time from 8 s until the end, special walk with the forearm at 0°. Figure 8-bottom shows that the oscillations induced by walking originate a variation in the vertical angle α of ±10°. This level is very low and there is no need to filter this oscillation to establish the operating mode of the electronic white cane. Additionally, the detection of the peaks and valleys of this oscillation can be used to confirm that the user is walking and estimate the distance (if the user’s stride is known to the system). In the future, this information of the accelerometer will be complemented with additional gyroscopes to perform some basic odometry of the user’s displacement and also some basic mapping of the obstacles found. This mapping feature can be very useful in repetitive daily displacements such as “going to work” or “returning home” where most of the relevant obstacles have a fixed location in the walking path.

### 3.2. Floor Mode Operation

**Figure 9.**
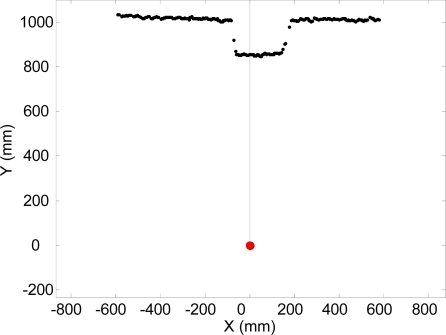
Example of LIDAR scan obtained in floor mode with an obstacle in the walking path.

The floor mode of the electronic white cane is started when a static user places its forearm with a vertical inclination α (measured with the accelerometer) of approximately 45° and with a horizontal orientation β of approximately 0°. The main objective of the floor mode is to explore the ground surface in front of the user while walking, detecting the floor and any obstacles or discontinuities. To this end, only a small angular range (covering approximately 1 m width) of the full scan range (−135° to 135°) acquired by the LIDAR (Figure 9) are first analyzed and then mapped into the tactile belt. The angular range was obtained by a trial an error process to reduce the cognitive load of the tactile information supplied to the user.

**Figure 10.**
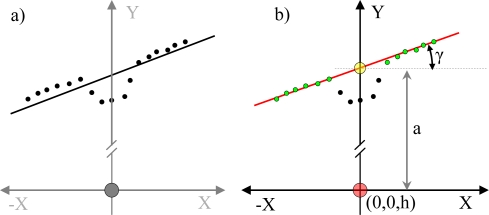
Representation of the procedure used to estimate the frontal line: **(a)** line obtained with all scan points, **(b)** final line obtained with the outliers removed.

Figure 9 shows an example of raw LIDAR data acquired in floor mode. The intersection between the scan plane and the ground plane defines a frontal line that can be computed as follows: (i) fitting a line applying a weighted LMS procedure to the selected points of the scan (more weight as the distance increases) [Figure 10(a)], (ii) comparing the scan points with the fitted line and classifying as outliers the points at ±100 mm from the line, (iii) if there are no outliers, this procedure ends, otherwise the outliers are deleted and the procedure is started again [Figure 10(b)]. Any obstacle or alteration in the floor in front of the user breaks (see Figure 9), shortens, rotates or displaces the location of this frontal ground line. For example, a big hole (as down stairs) in the walking path moves away the frontal line.

**Figure 11.**
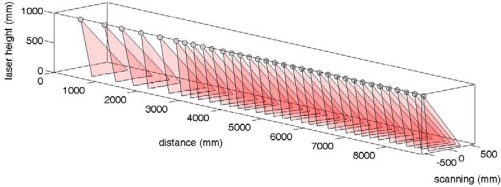
Three-dimensional representation example of the LIDAR scan plane in a forward walking.

As an example, Figure 11 shows a three-dimensional representation of an electronic white cane operating in floor mode during a forward walking displacement of approximately 9 meters at an average speed of 3 km/h. The grey sphere indicates the position of the LIDAR and the semitransparent red triangles indicate the orientation of the scan plane of the LIDAR and the intersection with the ground plane.

**Figure 12.**
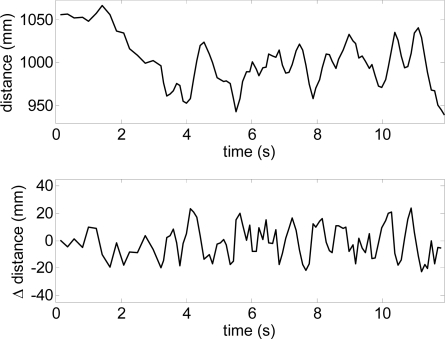
Distance user-frontal line obtained in the experiment of Figure 11.

**Figure 13.**
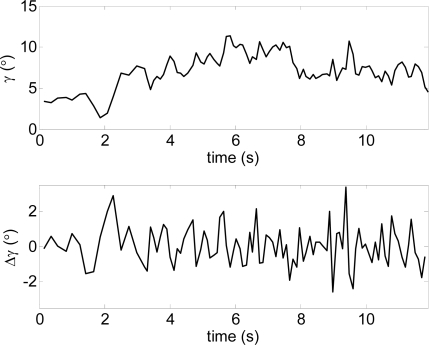
Inclination of the frontal line γ corresponding to the experiment of Figure 11.

Figure 12 shows the estimate of the distance to the frontal line (intersection between the scan and floor planes) and Figure 13 the estimate of the relative inclination γ [see Figure 10(b)] extracted both from the trajectory raw data represented in Figure 11. The involuntary bouncing of the arm while walking is projected into the estimate of the distance (Figure 12-top) but not into the estimate of the relative inclination. The differences between consecutive scans (Figure 12-bottom and Figure 13-bottom) show very small instantaneous values during a normal walk and this evolution can be used to detect sudden changes into the ground such as originated by big objects (walls or up stairs) or big holes (down stairs).

**Figure 14.**
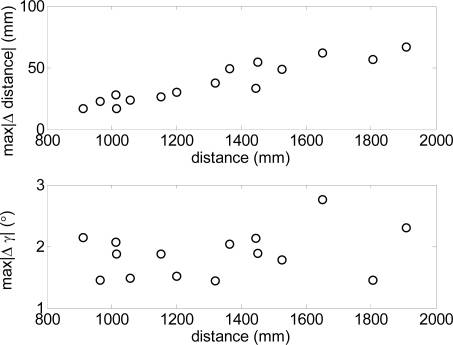
Maximum incremental values of the distance (top) and relative angular orientation (bottom) of the frontal line for different distance ranges.

To this end, Figure 14 shows the absolute maximum values of both incremental parameters obtained in different walking experiments relative to the average distance range explored by the electronic white cane. In such experiments the maximum increment of the distance (Figure 14-top) and relative angular orientation (Figure 14-bottom) was lower than 40 mm and 5° respectively when the distance range covered by the electronic white cane was between 1 and 1.2 m. These reference values can be used as a threshold to detect abnormal changes into the floor. Large exploratory ranges will require the definition of a linear adaptive threshold rather than a fixed value. Future electronic white cane implementations will include a small regulator dial next to the LIDAR to modify both thresholds without any additional equipment or configuration procedure.

**Figure 15.**
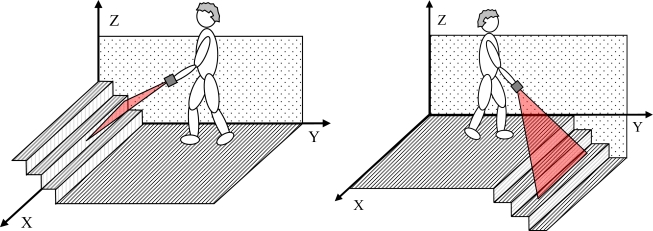
Up (left) and down (right) stairs detected with the electronic white cane.

**Figure 16.**
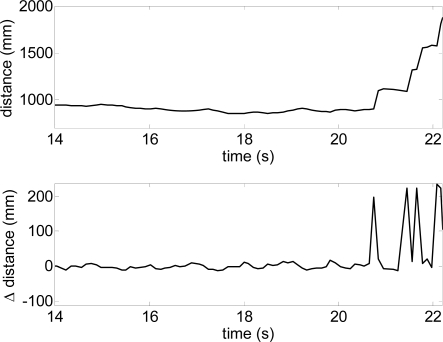
Distance to the frontal line obtained in one down stairs experiment (Figure 14-right).

Figure 15 shows a representation of one user in front of some upwards and downwards stairs. Figure 16 shows the evolution of the estimated distance to the frontal line and the difference between consecutive scans obtained when reaching a down stairs. In this case, the distance to the frontal line increases suddenly over passing the absolute threshold of 40 mm defined previously to detect obstacles in the walking path. Alternatively, Figure 16 shows the results of a similar up stairs experiment. In this case, the distance to the frontal line decreases suddenly on reaching the stairs, over passing again the absolute threshold of 40 mm previously defined. Table 2 summarizes some other typical situations expected.

The first (up) row of Table 2 corresponds to clear ground without obstacles. In this case, the computed frontal line (red line) has no outliers (the threshold limit of the outliers is represented by a dotted black line). The second row of Table 2 shows one object in the user’s path. In this case, the distance to the frontal line does not change but there are a large number of outliers (big dark points) breaking the line. The third row of Table 2 corresponds to the detection of one person in the user’s path. In this case, the frontal line has two breaks with a large number of outliers (big dark points). The fourth row of Table 2 corresponds to the appearance of one wall in the user’s path. In this case, the outliers (big dark points) always appear on the left or right of the frontal line (red line). In all cases, the relative position of the outliers is mapped into the user’s belt as tactile information, simulating the effect of the whiskers. Any other big obstacle in the user’s trajectory (stairs, frontal wall) will change suddenly the distance to the frontal line and will activate all actuators of the belt.

### 3.3. Frontal Mode Operation

The frontal mode of the electronic white cane is activated when the vertical inclination of the forearm α is approximately 90° and the horizontal inclination β approximately 0°. The main objective of the frontal mode is to explore the environment in front of the user while staying static, only moving the arm to complete the exploration. Again, only a small range of the complete scan range of the LIDAR was analyzed and mapped into the belt to reduce the cognitive load of the tactile information provided to the user.

**Figure 18.**
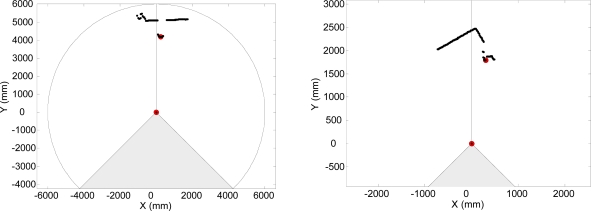
Example of partial scans analyzed in frontal mode. The closest obstacle (left at 4,188 mm, right at 1,795 mm) is located and labeled with a red circle.

Figure 18 shows two example scans obtained when the electronic white cane was operating in the frontal mode. The objects found are grouped and mapped directly as different pressure levels on the belt simulating the detection effect of the whiskers. In all cases, the maximum pressure level represents the closest obstacle/object without any additional processing. The measurement range and tactile pressure representation will be modified easily by the user in the final electronic white cane implementation.

### 3.4. Tactile Belt

**Figure 19.**
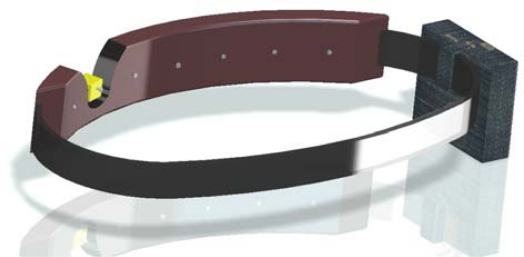
Representation of the tactile belt with seven miniature actuators and the control box.

The main objective of the tactile belt is to generate pressure information on the skin of the user’s waist. The tactile belt simulates the detection effect of the different hairs composing the virtual whisker. Figure 19 shows a CAD representation of one possible final implementation of the tactile belt with a detail showing one actuator (see Figure 2) and carrying the control box with the batteries and embedded electronics. The tactile information can be generated either by modulating the effective continuous pressure applied or modulating its frequency that is the proposed default operating method. It must be noticed that the sensitivity to tactile feedback changes when a user starts to move so the number of actuators (and thus the number of hairs of the whisker) was set at seven by a trial and error process. In the future, this apparently simple tactile interface will be studied in depth and evaluated with blind users for further improvements.

## 4. First Validation Results

The first validation results were obtained with non blind voluntary users. Table 3 summarizes the results obtained when operating the electronic white cane in floor mode. Obstacles and stairs were successfully detected in all tests performed, but the basic classification performed of the obstacle detected, based on the shape of the dynamic evolution of the distance to the frontal line needs further improvements.

When operating in frontal mode, the users reported that they could detect the environment and have a clear idea of what was close and far. Finally, the users suggested some improvements, the more relevant of which were:

- The inclusion of a reference tactile signal showing that the system is operating properly. When working in floor mode, no tactile information was given if the floor was correctly detected without any obstacles. Then, after several seconds of tactile silence, some users make erratic forearm movements to obtain some feedback from the system. As a consequence of this suggestion, the first and last actuators are activated sequentially to indicate that the system is working properly when nothing was detected.
- The inclusion of some voice description of relevant elements of the surrounding environment such as “door on the left”, “open door in front”, “wall on the right”, etc. when working in ground mode. This suggestion was very interesting and probably defines future evolutions of this electronic white cane proposal and requires the development of new processing algorithms applied to the raw data from the LIDAR.
- The implementation of a non-contact tracking mode to follow a moving person at a fixed distance while detecting obstacles in the path.
- The possibility of reducing the scanned angle when operating in the frontal mode, using the arm movement to complete the exploration of the environment.
- The inclusion of a small set of buttons to change between predefined settings or functional options.

## 5. Conclusions

This work proposes a new implementation of an electronic white cane inspired by animals’ whiskers and show the first results obtained with non blind users. This proposal is based on the combined use of a LIDAR, a tri-axial accelerometer, and a tactile belt. The experiments performed have shown that the information obtained with the accelerometer can be used to switch between floor mode, if the forearm (and the LIDAR) is pointing at the ground (α ≈ 45°), and frontal mode, if the forearm is parallel to the ground (α ≈ 90°). The main objective of the floor mode is the exploration of the ground surface just in front of the user while walking, ensuring safe walking and revealing the position of any obstacles or discontinuities in the current path. The main objective of the frontal mode is the exploration of the environment in front of the user while staying static, only moving the arm to complete a three-dimensional exploration. When working in floor mode, the intersection between the scan plane and the floor defines a frontal line. The presence of outliers in this frontal line reveals the existence of small objects in the walker’s path whereas the sudden change in the distance to the frontal line reveals the existence of big obstacles as walls or stairs. When operating in frontal mode, the results have shown that the user can have a clear idea of the surrounding environment up to a range of 6 m by moving the arm to complete the three-dimensional exploration. In all cases a tactile belt was used to show the information to the user simulating the effect of whisker with a reduced number of hairs.

As a future work, the proposed bioinspired electronic white cane will be tested with blind users to get specific functional feedback and evaluate and improve the effectiveness of the proposed detection algorithms with end users. Future research in this field will be focused in the incorporation of additional sensors and in the development of specific processing algorithms to improve the description of the environment and include alternative high-level descriptive warning advices.

## Figures and Tables

**Figure 17.**
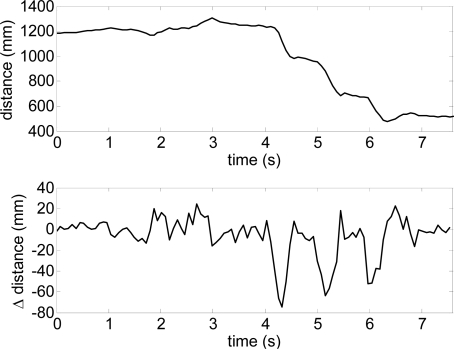
Distance to the frontal line obtained in one up stairs experiment (Figure 14-left).

**Table 1.** Detail of different vertical inclinations measured with the accelerometer when raising the user’s forearm.

Case A		Case B		Case C		Case D	
real	estimated	real	estimated	real	estimated	real	estimated
30.8°	32.5°	71.0°	70.6°	109.3°	109.2°	129.5	129.9°
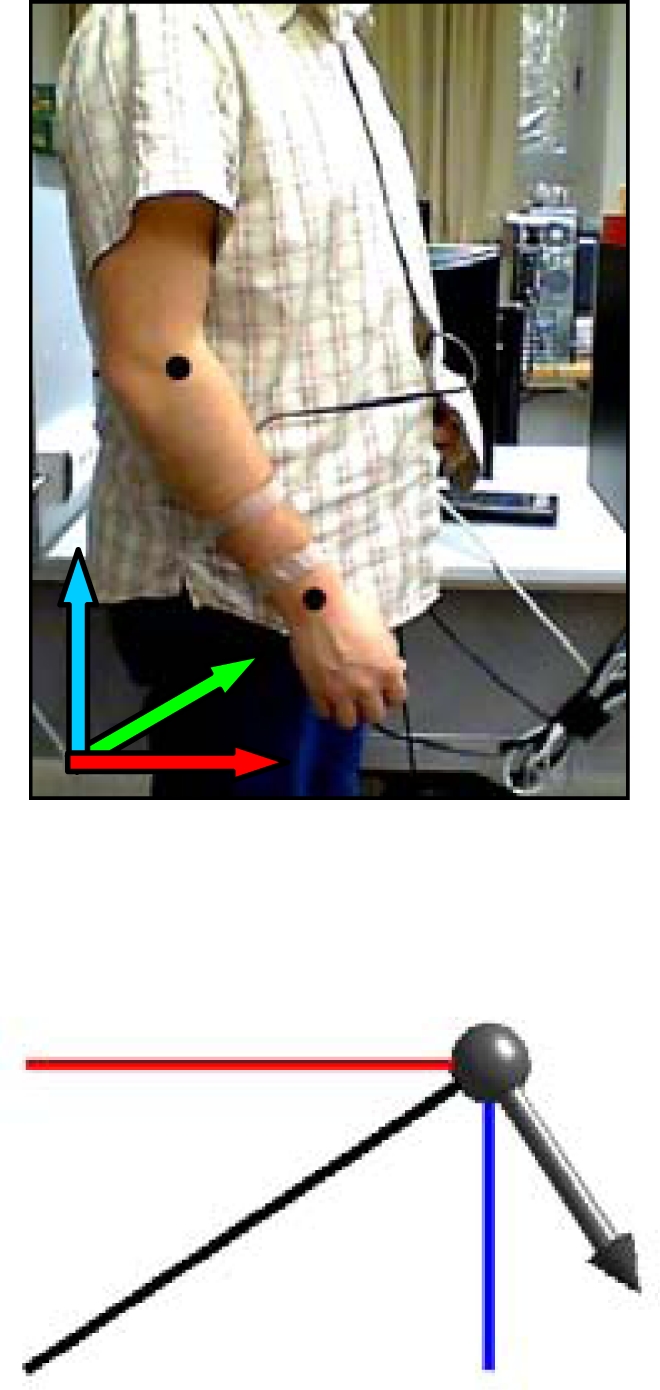		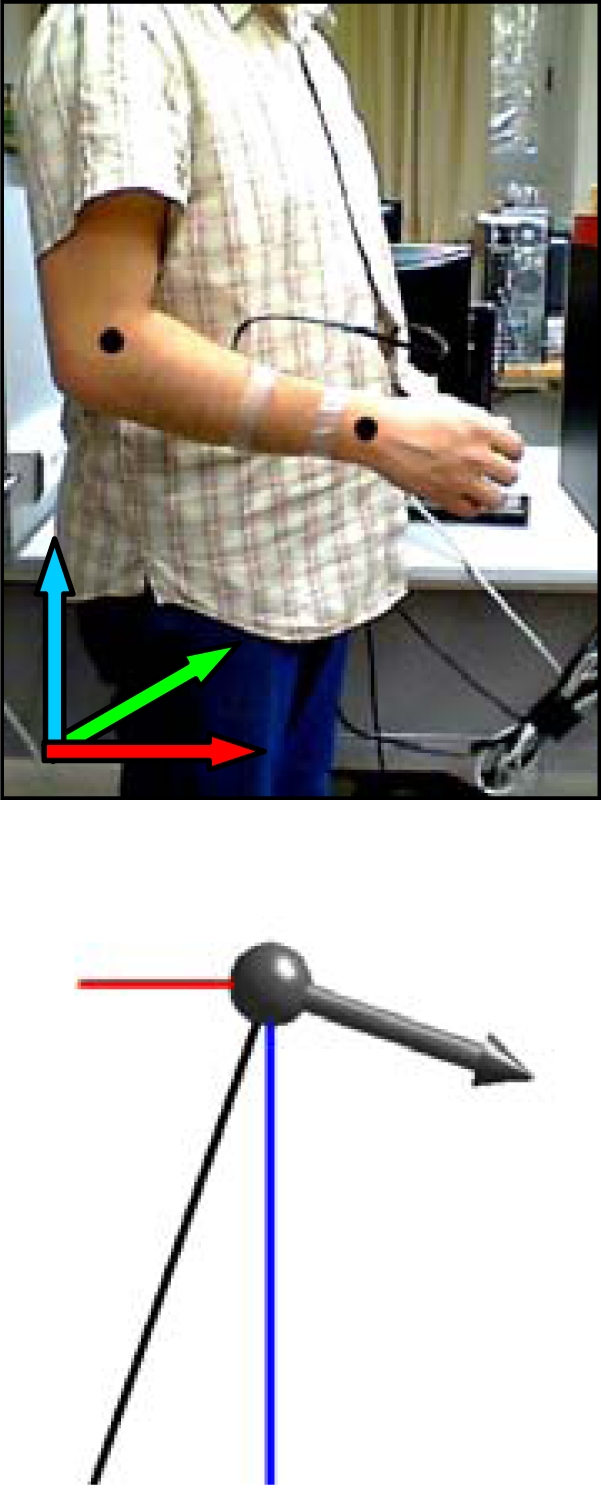		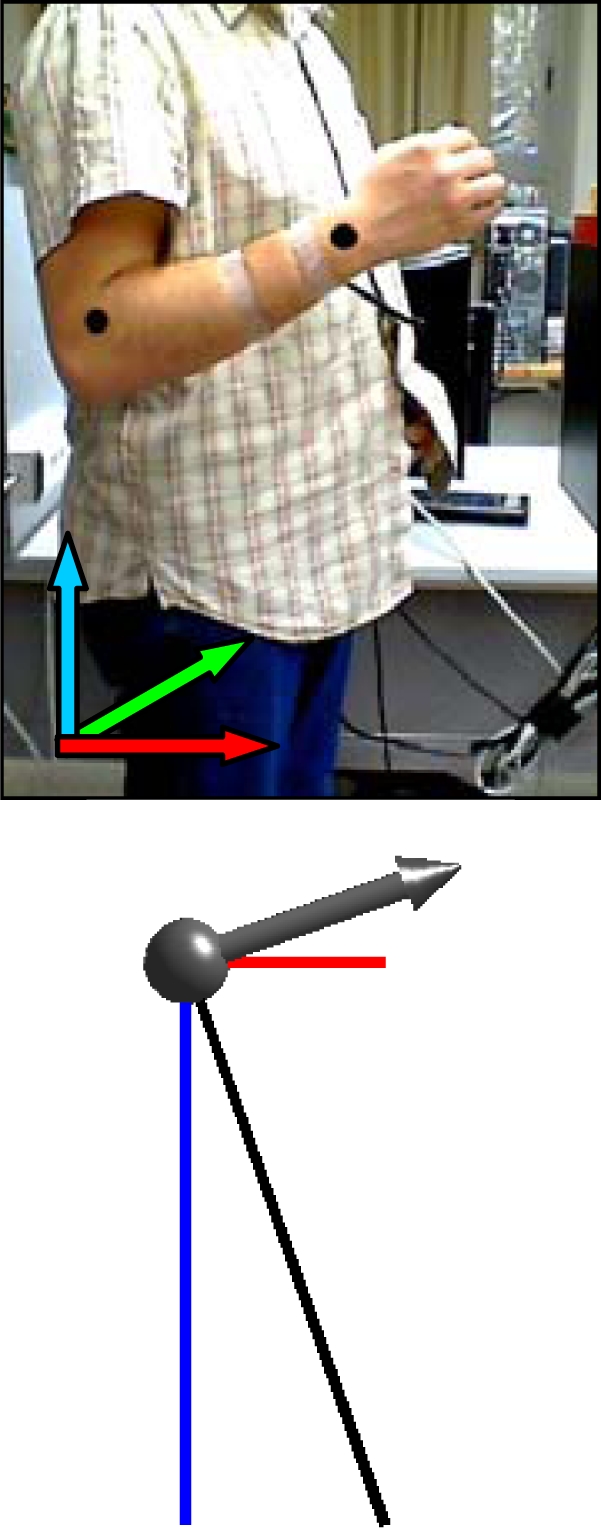		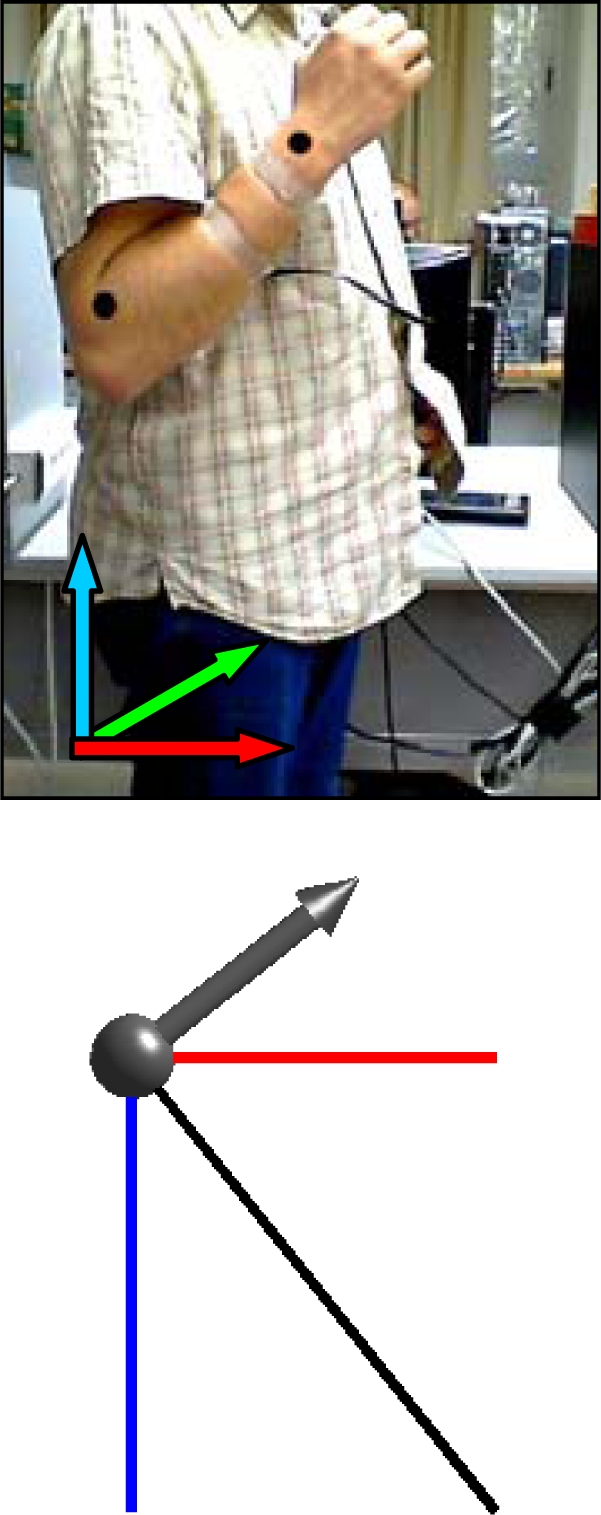	

**Table 2.**
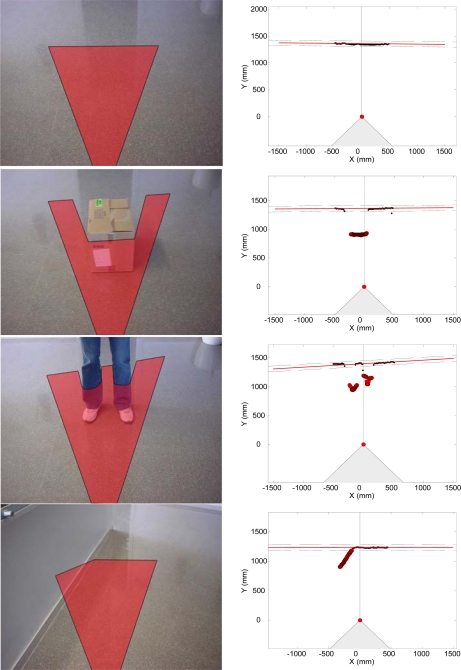
Images of four events (left) and its detection through the analysis of the frontal line (right).

**Table 3.** Detection results of the electronic white cane operating in floor mode.

		**Detected as (%)**			
		**Plain floor**	**Obstacle**	**Stairs Up**	**Stairs Down**
Plain floor		93	0	7	0
Stairs	Up	0	5	95	0
	Down	0	2	0	98
Cubic obstacle (side mm)	100	0	100	0	0
	200	0	100	0	0
	300	0	100	0	0
	400	0	98	2	0
	500	0	16	84	0
